# Nurses' Patient-Centeredness and Perceptions of Care among Medicaid Patients in Hospital Obstetrical Units

**DOI:** 10.1155/2013/563282

**Published:** 2013-08-20

**Authors:** Stephen J. Aragon, Liana J. Richardson, Wanda Lawrence, Sabina B. Gesell

**Affiliations:** ^1^Winston-Salem State University, School of Business and Economics, Department of Management and Marketing, Healthcare Administration Program, Winston-Salem, NC 27110, USA; ^2^University of North Carolina at Chapel Hill, Department of Sociology and Carolina Population Center, 155 Hamilton Hall, CB No.3210, Chapel Hill, NC 27599, USA; ^3^Winston-Salem State University, School of Health Sciences, Division of Nursing, Winston-Salem, NC 27110, USA; ^4^Wake Forest School of Medicine, Public Health Sciences, Department of Social Sciences and Health Policy, and Maya Angelou Center for Health Equity, Winston-Salem, NC 27157, USA

## Abstract

*Objective*. This study examined to what degree patient-centeredness—measured as an underlying ability of obstetrical nurses—influenced Medicaid patients' satisfaction with care in hospital obstetrical units. * Design*. Multigroup structural equation modeling design, using three cross-sectional random samples (*n* = 300 each) from the 2003 Press Ganey National Inpatient Database. *Setting*. Self-administered mail surveys. *Participants*. 900 Medicaid recipients recently discharged from inpatient hospital obstetrical units across the United States. *Methods*. Multigroup structural equation modeling was used to test the goodness of fit between a hypothesized model based on the Primary Provider Theory and patients' ratings of nurses. *Results*. The model fitted the data well, was stable across three random samples, and was sustained when compared to a competing model. The patient-centeredness of nurses significantly influenced overall patient satisfaction and explained 66% of its variability. When nurses' patient-centeredness increased by one standard deviation, patients' satisfaction increased by 0.80 standard deviation. *Conclusion*. This study offers a novel approach to the measurement of the patient-centeredness of nurses and a paradigm for increasing it and its influence on Medicaid patients' satisfaction in hospital obstetrical units.

## 1. Introduction

Although rates of maternal mortality are low in the United States [US], evidence is accumulating that the country's rates of maternal *morbidity* during labor and delivery are high [1] and rates of severe obstetric complications are increasing (e.g., hypertension [2], pulmonary embolism [3]). In addition, it is well known that the US ranks behind most other developed countries in its rates of adverse birth outcomes, such as low birth weight [4], preterm birth [5–7], and infant mortality [4]. Persistent racial and socioeconomic disparities in adverse birth outcomes [8] and maternal morbidity [9] and mortality [10] also have been well documented.

Obstetrical (OB) units in hospitals can be crucial points of intervention to prevent the negative consequences of maternal morbidity and especially conditions that give rise to early labor and delivery, such as hypertensive disorders. As in other healthcare settings, the provider-patient relationship in the OB unit is a central locus of communication, understanding, and delivery of care [11]. If women do not receive adequate information or care during their stay in an OB unit, it could be detrimental for their own health as well as the health of their infants. High-quality care is, therefore, essential for maternal and infant health in OB settings [12]. 

During the past decade, patient-centeredness has been widely acknowledged as a key component of high-quality patient care by the US Institute of Medicine [13] and the US Agency for Healthcare Research and Quality [14, 15]. Previous studies have revealed positive benefits of patient-centeredness for patients, such as increased participation in the clinical encounter [16], trust in their providers [17], and satisfaction with care [16]; better adherence to treatment recommendations [18]; and improved health outcomes [16–18]. Most investigations of patient-centeredness, however, have focused primarily on the encounter between *physicians* and patients. This emphasis ignores the very important role of *nurses*, particularly in OB units where they are heavily involved in educating, coaching, assisting, and providing routine care to women during labor, childbirth, and recovery. In addition, a recurring methodological shortcoming in the literature on patient-centeredness is the predominant focus on assessing the independent effects of individual care behaviors (e.g., listening carefully, explaining things clearly, or spending enough time with patients) rather than viewing these behaviors as reflection of a single underlying multivariate construct, that is, a latent variable representing a trait (versus a state) of healthcare providers. As a result, a generally accepted measurement model of patient centeredness is non-existent, and current operational definitions lack sufficient specificity, as described in detail elsewhere (e.g., [19, 20]).

*Call  Out  1.* Many investigations of patient-centeredness focus primarily on the encounter between physicians and patients, ignoring the important role of nurses particularly in hospital OB units.

**Figure 1:**
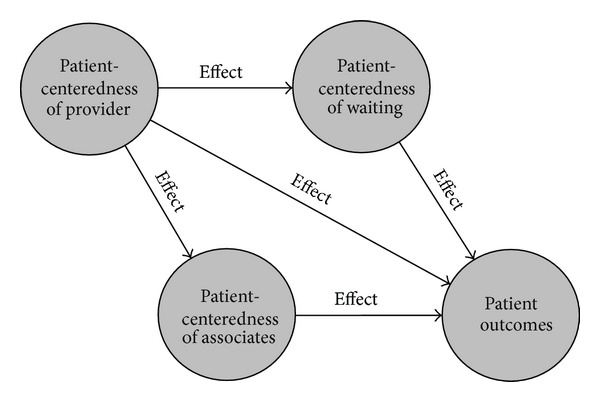
Primary Provider Theory. (Adapted from Aragon [19, 21, 23].)

In this study, we examined whether patient-centeredness is a measurable underlying ability of OB *nurses* and the extent to which it positively influences satisfaction with care among Medicaid patients in hospital OB units. We focused on Medicaid patients because of the greater burden of maternal morbidity and adverse birth outcomes borne by women of low socioeconomic status. We hypothesized a measurement model for the latent variable, patient-centeredness, to describe it with greater reliability than previous studies, while also accounting for potential measurement error. Additionally, we applied the Primary Provider Theory, a generalizable theory of how the patient-centeredness of healthcare providers affects patient outcomes (Figure 1).

As previously described by Aragon and colleagues (e.g., [19, 21]), the Primary Provider Theory holds that patient-centeredness is an underlying ability of healthcare providers that influences outcomes rather than just a list of discrete behaviors or processes generally subsumed by the term “patient-centered care.” Consistent with this proposition, the theory also holds that clinical competency is a necessary, but insufficient condition of healthcare quality and desired outcomes, because the delivery of healthcare necessarily requires the provider's interpersonal interaction with patients. According to the theory, patient-centeredness is reflected in a healthcare provider's approachability, interpersonal competency, respectfulness, concern (for patients' best interests, feelings, and needs), and lack of prejudice. It also reflects the provider's desire to communicate with and inform patients, as evidenced by an absence of domination over patients, in favor of encouraging their participation in the decision-making process. Finally, the theory holds that patients are the best judges of the patient-centeredness of their health providers.

**Figure 2:**
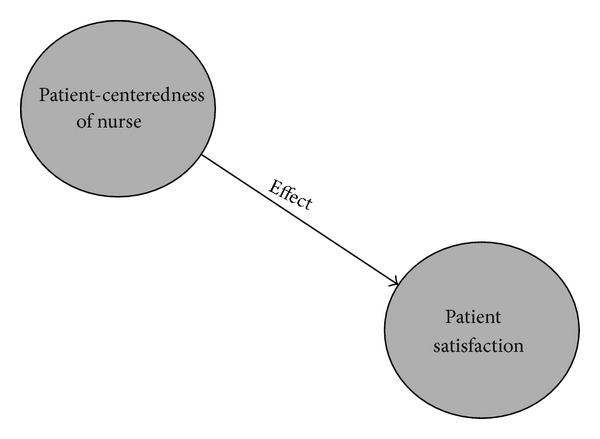
Study hypothesis.

A subproposition of the Primary Provider Theory is that the patient-centeredness of the primary provider's associates, in this instance nurses, also influences patient outcomes. Thus, in the present study, we hypothesized that a positive association between the patient-centeredness of OB nurses and Medicaid patients' satisfaction would be observed (Figure 2). Accordingly, an increase in the patient-centeredness of OB nurses was expected to be associated with improvement in satisfaction with care among Medicaid OB patients.

## 2. Methods

### 2.1. Participants

All participant data came from the Press Ganey National Inpatient Database, which consists of data from over 1,000,000 recently discharged patients from over 1,000 hospitals across 49 states. The participant sampling frame was limited to females, Medicaid recipients, in 2003, who received care in one of the 730 inpatient OB units found in the hospital sample and who had full information on the variables of interest. From this pool of 1,770 eligible respondents, we randomly selected three independent cross-sectional samples of 300 patients each for analysis (overall  *N* = 900). 

### 2.2. Instrument

All data for the present study was collected with the Press Ganey National Inpatient Survey, a 49-question standardized, mail survey designed to measure the care experiences of patients discharged from US hospitals for continuous quality improvement purposes. Responses to all items are scored by respondents on a balanced 5-point scale ranging from very poor (1) to very good (5). Evidence of the instrument's internal consistency (*α* = 0.98) and construct validity has been documented elsewhere [22], as has its use in measuring patient experiences across a variety of healthcare settings, such as emergency departments [23], primary care [19, 24], outpatient cancer care [25], and assisted living facilities [26]. 

### 2.3. Data Collection

Surveys were mailed throughout the year to random samples of recently discharged patients, with a postage-paid return envelope and a cover letter assuring confidentiality. Continuous sampling was employed to reduce seasonal variation. Typically received within five days of hospital discharge, surveys were mailed within the six weeks recommended by the current research on reliability of patient responses [27]. Automated mailing reduced the possibility of selection bias by ensuring that all patients chosen by a hospital's sampling logic received a survey. Completed surveys were coded and entered into the 2003 Press Ganey National Inpatient Database. 

### 2.4. Measures

#### 2.4.1. Patient-Centeredness

We used four items from the 2003 Press Ganey National Inpatient Survey as indicators of the latent variable *patient-centeredness*: the nurse's friendliness and courtesy (N1), how well the nurse kept his/her patients informed (N5), the nurse's attitude toward patients' requests (N3), and the attention nurses paid to their patients' personal needs (N4).

#### 2.4.2. Patient Satisfaction

We used two items from the survey as indicators of the latent variable *patient satisfaction*: likelihood of recommending the hospital to others (O3) and their overall rating of care at the hospital (O4).

#### 2.4.3. Control Variables

We treated *patient's age* (age) and *length of stay* (days) as potential confounders in all analyses.

### 2.5. Analytical Methods

**Figure 3:**
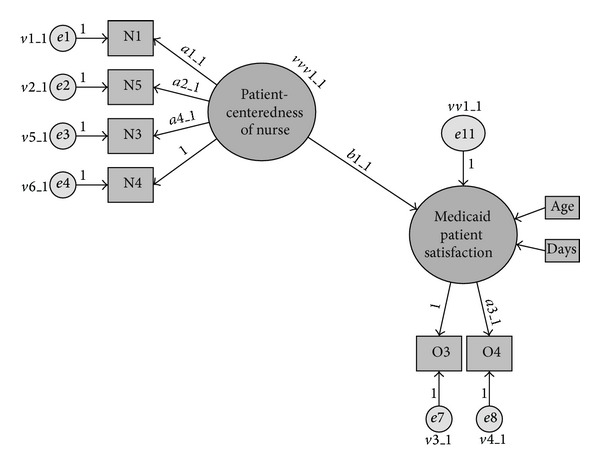
Hypothesized structural model. Key: e1–e4, e7-e8: error variances for each of the observed variables. a1_1, a2_1, a4_1, a3_1: factor loadings (i.e., regression weights). b1_1: regression weight (i.e., coefficient for the association between patient-centeredness and satisfaction). e11: disturbance (i.e., unexplained variance) for the latent variable, patient satisfaction.

First, confirmatory factor analysis (CFA) was used to confirm the reliability and validity of the study's measurement model. We then used multigroup structural equation modeling, with asymptotic distribution free estimation, and cross-group equality constraints to test the hypothesized structural model (Figure 3) and its stability across the three independent random samples of Medicaid OB patients. The model's trustworthiness was based on a convergence of evidence, including its empirical fit, stability across samples, sustainability when compared to a rival model, and consistency with the Primary Provider Theory. The model was judged to be well fitting if its chi-square test statistic was not statistically significant (*χ* ^2^ = ns), root mean square error of approximation (RMSEA) was less than or equal to 0.05, and comparative fit index (CFI) was greater than or equal to 0.90. A chi-square difference test was used to test the stability of the model across the samples and to compare the model with its effects constrained to equality across all three samples and a competing model with unequal effects. A nonsignificant chi-square difference test statistic (*χ* ^2^Δ = ns) indicated that the constrained model provided a better fit to the data.

*Note.* Structural equation modeling (SEM) is a flexible multivariate methodology for simultaneously estimating population values of directed relationships (arrows/effects) among unobserved (circles) and observed (squares) variables, in complex models. It is computationally intensive and employs path analysis, matrix algebra, confirmatory factor analysis, multiple regression, and nonlinear optimization. Its models are based on the assumption that a model produced covariance matrix (the model world) equals the sample covariance matrix (the real world). After a model is estimated, its reproduced covariance matrix is compared to the sample covariance matrix to assess its empirical fit. This assessment either falsifies or supports the model.

## 3. Results

### 3.1. Descriptive Statistics

The three random samples selected for analysis were similar in age and length of stay in the hospital, as well as their reports of nurses' patient-centeredness and their satisfaction with care (Table 1). For all variables in the study, mean differences across the three samples were less than 0.25.

### 3.2. Multivariate Results

#### 3.2.1. Measurement Model

Overall fit statistics suggested that the measurement model for the two latent variables in our conceptual model provided adequate fit to the data (*χ* ^2^ = 54.64,  df = 51,  *P* = 0.34; RMSEA = 0.01; CFI = 0.98). In addition, all factor loadings for the indicators of the two latent variables were large in magnitude and statistically significant at the *P* < 0.001 level, with high squared multiple correlation (*R* ^2^) values (Table 2). With a composite reliability (CR) of 0.935 and average variance extracted (AVE) of 0.782, our patient-centeredness measure exceeded recommended reliability and convergent validity thresholds of 0.70 and 0.50 [28]. Similarly, patient satisfaction's CR and AVE were 0.922 and 0.855, respectively, thereby supporting the reliability and validity of the measure.

#### 3.2.2. Structural Model

**Figure 4:**
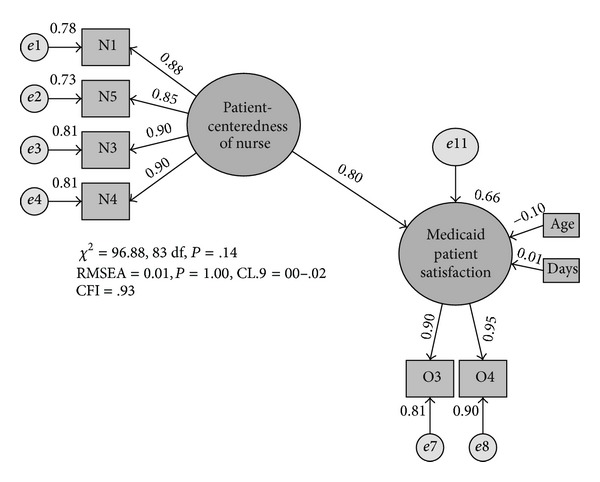
Standardized effects. (All paths in the model statistically significant at the *P* < 0.001 level, except for the paths between age and patient satisfaction and between days (i.e., length of stay) and patient satisfaction (*P* > 0.05 for both variables).)

Evidence from multigroup analysis of the model across the three samples converged in support of the model's fit (*χ* ^2^ = 96.88, df = 83, *P* = 0.14; RMSEA = 0.014; CFI = 0.927; Figure 4). As hypothesized, nurses' patient-centeredness significantly influenced Medicaid OB patients' satisfaction with care, removing the influence of patient's age and length of stay. Specifically, when nurses' patient-centeredness increased by one standard deviation, Medicaid OB patients' satisfaction increased by 0.80 standard deviation (*P* < 0.001). Moreover, nurses' patient-centeredness accounted for 66% of the overall variability in Medicaid OB patients' satisfaction. This pattern of findings held and effects were stable across all three samples of Medicaid OB patients. Moreover, the model with effects constrained to equality across all three samples was sustained when it was compared to a competing model with unconstrained or unequal effects across the samples (*χ* ^2^Δ = 27.03,   df = 26, *P* = 0.408).

## 4. Discussion

In this study, we examined whether and to what degree the patient-centeredness of obstetrical nurses influenced satisfaction with care among Medicaid patients in hospital OB units. We found that when operationalized as a multivariate latent construct representing an underlying ability of nurses patient-centeredness significantly influenced overall Medicaid OB patients' satisfaction, explaining a preponderance of its variability, a finding that held true across three independent random samples and despite adjustment for patient age and length of stay. Evidence supporting the construct validity and reliability of the model included its empirical fit, which was better than that of a competing model, its replication across three random samples, and its concordance with the Primary Provider Theory. Additional evidence supporting the model's trustworthiness included the magnitude and strength of its effects across samples and the high squared multiple correlations of its latent variables, which represent lower bounds of reliability. Thus, the weight and convergence of the evidence supported the inferences of our hypothesized model. 

*Call Out 2.* When operationalized as a multivariate latent construct representing an underlying ability of nurses, patient-centeredness significantly influenced patients' satisfaction and explained most of its variability.

The results of this study are consistent with the findings of Ruiz-Moral et al. [16], who found greater satisfaction among patients who felt that their encounters with healthcare providers were more patient-centered [16]. To our knowledge, however, this is the first investigation of this relationship that treats and measures patient-centeredness as a latent ability of health nurses. 

Several limitations of the study are worth noting. While there is no generally accepted response rate in survey research, patients who respond to surveys could be different from those who do not [29]. For example, minority patients could be underrepresented if they are less likely to return a completed survey [30, 31]. However, potential impact of this possibility was indeterminable because the dataset does not contain information on race/ethnicity. Finally, there are an infinite number of behaviors that could conceivably be reflective of the latent patient-centeredness construct. We used those, which correlate with patient-centeredness as conceptualized by the Primary Provider Theory, giving us an advantage over previous atheoretical operationalizations of the construct [20].

*Call Out 3.* The study's results suggest that the Primary Provider Theory can be used to improve OB nursing education and develop interventions for improving patients' care experiences.

Notwithstanding these limitations, this study offers an alternative paradigm for measuring and improving the patient-centeredness of nurses and the satisfaction of Medicaid obstetrical patients. In addition to healthcare quality improvement, the model has implications for OB nursing education, certification, and licensing, as well as interventions to improve patient satisfaction with care. The results of this study suggest, for example, that hospitals' efforts to improve Medicaid patients' satisfaction should place emphasis on patient-centered care behaviors, like friendliness and courtesy, keeping patients informed, being positive about patients' requests, and paying attention to patients' special requests. 

Further research is recommended with larger and more diverse further research is needed with larger and more diverse samples of OB patients that will allow examination of subgroup differences by race/ethnicity. As noted in the introduction, we view the receipt of adequate information and care of mothers during their stay in hospital OB units as patient-centered care critical to their health critical to their health as well as the health of their offspring. Thus, extending the model beyond patient satisfaction to include more wide-reaching effects on noncognitive patient outcomes is also suggested. Taken together such analyses will help improve our understanding not only of how nurses' patient-centeredness impacts OB patients of low socioeconomic status, such as the women included in our study, but also what role OB nurses can play to help improve care experiences and ultimately reduce socioeconomic and other disparities in maternal morbidity, mortality, and adverse birth outcomes.

## Figures and Tables

**Table 1:** Sample characteristics.

Variable	Sample_1_ (*n* = 300)		Sample_2_ (*n* = 300)		Sample_3_ (*n* = 300)	
	Mean	SD	Mean	SD	Mean	SD
Age	24.8	7.02	24.81	5.44	25.1	6.06
Length of stay	2.93	1.28	2.82	1.11	3.04	1.92
Friendliness and courtesy (N1)	4.65	.690	4.57	.762	4.55	.728
Attitude towards requests (N3)	4.58	.729	4.50	.832	4.46	.815
Attention to special needs (N4)	4.54	.819	4.48	.803	4.46	.760
Kept patient informed (N5)	4.49	.816	4.42	.872	4.41	.855
Likelihood to recommend (O3)	4.60	.750	4.62	.738	4.51	.729
Overall rating of care (O4)	4.62	.686	4.58	.752	4.50	.828

**Table 2:** Measurement model statistics^a^.

			Regression weights		S.E.	*R* ^2^	C.R.
			Unstandardized^b^	Standardized^b^			
N1	←	Patient-centeredness of nurse	0.89	0.89	0.038	0.78	23.103
N3	←	Patient-centeredness of nurse	0.98	0.90	0.037	0.82	26.645
N4	←	Patient-centeredness of nurse	1.00^c^	0.90		0.81	
N5	←	Patient-centeredness of nurse	1.02	0.84	0.029	0.71	34.832
O3	←	Patient satisfaction	1.00^c^	0.91		0.83	
O4	←	Patient satisfaction	1.01	0.95	0.026	0.91	38.874

Abbreviations: S.E.: standard error; *R* ^2^: squared multiple correlation; C.R.: critical ratio.

^a^A single measurement model was estimated with a correlation between the two latent variables (*r* = 0.83).

^b^All regression weights (i.e., factor loadings) were significant at *P* < 0.001; two-tailed tests.

^c^Parameter constrained to 1.000 to scale the construct.
